# Laparoscopic selection for surgery in epithelial ovarian cancer. A short review

**DOI:** 10.52054/FVVO.15.1.060

**Published:** 2023-03-31

**Authors:** v Ghirardi, A Fagotti, G Scambia

**Affiliations:** Division of Gynaecologic Oncology, Fondazione Policlinico Universitario A. Gemelli - IRCCS, Rome, Italy; Universita Cattolica del Sacro Cuore, Rome, Italy.

**Keywords:** Ovarian cancer, laparoscopy, selection, surgery

## Abstract

The role of laparoscopy as a treatment selection method in ovarian cancer patients is receiving growing attention in surgical practice in both early and advanced-stage disease. When the disease is confined to the ovary, intraoperative laparoscopic assessment of the tumour features is needed to select the best surgical approach in order to prevent intraoperative spillage of cancer cells which would negatively impact patient prognosis. The role of laparoscopy as a disease distribution assessment tool in cases of advanced-stage disease is now accepted by current guidelines as an effective treatment strategy selection. Indeed, a published and validated laparoscopic scoring system, based on laparoscopic assessed intra-abdominal disease dissemination features have been demonstrated to be a reliable predictor of optimal cytoreduction achievement. This subsequently reduces the exploratory laparotomy rate in both primary and interval debulking surgery setting.

Furthermore, in cases of recurrent disease, the use of laparoscopy to predict whether complete tumour resection can be achieved is accepted by available guidelines. In this setting, the combination of laparoscopy and imaging techniques to manage platinum sensitive recurrent ovarian cancer cases showed a high accuracy in appropriately selected patients for secondary cytoreductive surgery.

In this article we describe the role of laparoscopy in the treatment selection-process in ovarian cancer patients.

## Introduction

The multiple benefits of laparoscopy as minimally invasive surgical approach in gynecologic oncology had been widely demonstrated.

As ovarian cancer is the most lethal among gynecological cancers (Vergote et al., 2022) and because treatment strategy has to be planned in relation to multiple variables, the potential of a minimally invasive approach in order not only to treat but also to select adequate cure planning has been gaining more and more attention over the last few decades.

Indeed in both advanced and early-stage disease, choosing the adequate treatment strategy appears to be strongly associated with patient’s oncologic outcomes. On this topic, the role of laparoscopy as one of the most valuable treatment selection tool is worth to be described throughout every step of ovarian cancer treatment journey (Table I).

**Table I:** Evidence on laparoscopy as a selection method for surgery in ovarian cancer.

Early stage disease	Primary debulking surgery (PDS)	Interval debulking surgery (IDS)	Recurrent disease
Ghirardi et al. 2022	Fagotti et al. 2006	Fagotti et al. 2009	Bizzarri et al.2022
	Petrillo et al. 2015		
	Hansen et al. 2018		
	Llueca et al. 2021		
	Vizzielli et al.2016		

## Laparoscopy in early-stage ovarian cancer

The use of a laparoscopic approach to surgically treat early-stage ovarian cancer in terms of feasibility and safety has been validated by multiple studies (Ghezzi et al., 2007; Park et al., 2008; Park et al., 2013). Indeed, it has been demonstrated to as be as safe and effective as laparotomy but with better postoperative outcomes (Park et al., 2008). In addition, as early-stage disease can often be diagnosed at a pre-menopausal age, the safety, adequacy, and successful fertility outcome of laparoscopic procedures in case of fertility sparing surgery have been previously described (Cromi et al., 2014; Ghezzi et al., 2016).

Evidence on the oncologic outcomes of early- stage ovarian cancer patients treated with minimally invasive surgery has been controversial. Despite the superimposable survival outcomes of laparoscopically treated patients compared to a similar group of women treated via laparotomy described by both Gallotta et al. (2016) and Park et al. (2008), results from a Cochrane review (Falcetta et al., 2016) stated that no good quality evidence is yet available to help quantify the benefit of laparoscopy for the management of early-stage ovarian cancer.

One of the main concerns on this subject is the association between laparoscopic surgery and intraoperative spillage of cancer cells. Specifically, whether performing surgery through a minimally invasive approach may increase tumour spillage rate and therefore affect patient’s survival (Vergote et al., 2001). It is well recognised that cancer spillage may result in upstaging of the disease from International Federation of Obstetrics and Gynecology (FIGO) stage IA-IB to 1C1 (Prat, 2015), and in doing so worsen the patient’s prognosis (Wei et al., 2017).

A recent large retrospective study (Matsuo et al., 2020) analysed the trends in minimally invasive surgery (MIS) use and capsule rupture on a cohort of over 8000 patients, together with the association between MIS, rupture rate and survival in early- stage ovarian cancer.

In their paper they demonstrated a 22.5% rate of capsule rupture among the population. There was an increasing trend in MIS use seen through the years as it gained more space in surgical practice. Importantly, they demonstrated that capsule rupture had a detrimental effect on survival (4-year overall survival 91.5% for MIS and non-ruptured tumours; 90.5% for open surgery and non-ruptured tumours, and 88.9% for MIS and ruptured tumours; 86.8% for open surgery and ruptured tumours, p = 0.001) and that that both the use of MIS and larger tumour size were independently associated with an increased risk of capsule disruption on multivariate analysis. It showed that both the use of MIS and larger tumour size were independently associated with an increased risk of capsule disruption on multivariate analysis.

Patients selection appears to be of utmost importance in case of early-stage disease, mostly to appropriately choose surgical approach. In order to provide an objective selection method on the appropriate surgical strategy in this group of patients, a recently published retrospective study (Ghirardi et al., 2022) aimed to identify pre-operative and intra- operative patient and tumour characteristics which were associated with an increased risk of tumour rupture during surgery through minimally invasive approach for early-stage ovarian cancer. Based on these findings, it aimed to create a scoring system to predict the risk of tumour rupture during MIS.

In this study univariate analysis demonstrated that both tumour diameter and adhesions to ovarian fossa peritoneum were independently associated with tumour rupture and therefore included in the scoring system designed to predict the likelihood of causing intraoperative cancer spillage.

With the leading aim to select patients suitable for MIS, this scoring system can represent a useful tool to help clinicians identify those cases in which MIS may represent a safe option.

## Laparoscopy in advanced stage disease

In cases of advanced stage disease, the role of laparoscopy in the primary treatment setting to assess disease distribution is well recognised. Indeed, in the most recent ESMO, ESGO consensus conference (Colombo et al., 2019) and NCCN guidelines (National Comprehensive Cancer Network, 2019) laparoscopy is accepted as a reliable predictor of tumour burden and also optimal cytoreduction.

With the aim to provide an objective and standardised disease assessment model, the Predictive Index Value (PIV) was designed in 2006 by Fagotti et al. (2006). In their study, 64 advanced ovarian cancer patients underwent laparoscopy and longitudinal laparotomy to define the chances of optimal debulking. Seven laparoscopic parameters were identified and associated to a numerical variable in relation to the strength of statistical association. In the final model, a predictive index score > or = 8 identified patients undergoing suboptimal surgery with a specificity of 100%. They described a positive and negative predictive and values of 100% and 70% respectively.

Together with the internal validation published in 2015 (Petrillo et al., 2015), the concordance of the scoring algorithm (Fagotti et al., 2006) with the intraoperative findings identified at primary debulking surgery (PDS) was retrospectively assessed in 226 patients who underwent both diagnostic laparoscopy and laparotomy exploration of the abdominal cavity. A 96% overall concordance between the two assessments was identified and laparoscopic assessment of the abdominal cavity was considered suitable to predict no gross residual disease (NGR) in advanced ovarian cancer (Hansen et al., 2018).

Other than the PIV score (Fagotti et al., 2006), few other laparoscopic predictive models have been designed and validated. The R3 and R4 model scores (Llueca et al., 2019) use computed tomographic, laparoscopic data and lesion size score to determine peritoneal cancer index (PCI). The scores of the above-mentioned models (Llueca et al., 2019) were evaluated in a retrospective study (Llueca et al., 2021) which included 103 advanced ovarian cancer patients. The study concluded that the three models, including laparoscopic assessment, were able to predict suboptimal cytoreductive surgery, but more reliable for predicting NGR.

Laparoscopic assessment aims to determine if complete cytoreduction is possible and, with under-vision biopsies, a histological tumour result can be sought. In many centres, upfront debulking surgery (whenever feasible) is scheduled a few days following disease assessment. A surgical assessment on the likelihood of achieving NGR at the end of surgery and a tumour histotype are both crucial in this interval decision making process. It has been demonstrated that some ovarian cancer histotypes, such as low grade serous and mucinous cancers, have a lower rate of response to neo-adjuvant chemotherapy compared to high grade serous disease (Grabowski et al., 2016; Sugiyama et al., 2000; Morice et al., 2019). In selected cases, upfront debulking surgery may be the best option with respect to survival despite a possible unfavourable tumour burden. Similar considerations can be made in cases of metastatic ovarian disease arising from a different primary tumour at final pathology report, where clinical presentation may be similar, but treatment pathway may significantly differ (Kubeček et al., 2017).

In centres where one-step procedure is an option and decision to proceed to upfront surgery is made from both laparoscopic assessment of disease burden and tumour histology from frozen section of the material, the presence of adequately trained pathologists represents a crucial aspect to prevent treatment pit-falls.

Laparoscopic assessment of tumour burden can also be used to estimate the risk of major post-operative complications after PDS. Indeed, a laparoscopic adjusted model that estimates post-operative morbidity was developed in 2016 (Vizzielli et al., 2016). This model uses the PIV (Fagotti et al., 2006) score, patients’ ECOG performance status, volume of ascites and CA125 levels as predictive variables.

Using these variables, the predictive model is able to provide an estimation of the major complication rate of specific procedures, which can provide useful information when planning treatment strategy.

The same selection process can be applied to the interval debulking surgery setting (IDS). Indeed in 2010, a modified PIV score (Fagotti et al., 2010) was prospectively created and published to help identify patients suitable for complete cytoreduction after neo-adjuvant chemotherapy (NACT). In this study, 4 of the already mentioned variables of the PIV score (Fagotti et al., 2006) were included in the final calculation, each of them scoring 2 points with the same statistical process. With a PIV (Fagotti et al., 2006) >4 the probability of optimally resecting the disease at interval debulking laparotomy is equal to 0 and therefore surgery should be abandoned.

## Laparoscopy in recurrent disease

Despite less available data than with primary treatment, secondary cytoreductive surgery (SCS) with laparoscopy is accepted by the NCCN guidelines (National Comprehensive Cancer Network: NCCN Clinical Practice Guidelines in Oncology: (NCCN Guidelines®): Ovarian Cancer (Version 4.2019), 2019) as an adequate strategy to assess if optimal cytoreduction is likely to be achieved.

Indeed, on this topic a triage algorithm to manage platinum sensitive recurrent ovarian cancer cases has been recently published (Bizzarri et al., 2022).

In this study, authors selected 272 recurrent ovarian cancer patients with both PET-CT scan and diagnostic laparoscopy. In the absence of extra- abdominal metastases at imaging, and unresectable miliary disease at laparoscopy, patients were selected for SCS. After the selection process, SCS was performed in 65.4% of the cases with complete gross resection achieved in 87.1%. Overall, an accuracy of over 90%21 selecting patients for SCS was demonstrated, regardless of the previous administered treatment (either PDS or NACT/IDS).

## Conclusions

In conclusion, in case of early-stage disease, selection of surgical strategy appears to be necessary in order to prevent intraoperative cancer spillage and negatively impact patient survival outcomes. On this topic, the application of the laparoscopic scoring system to estimate the risk of capsule disruption (Ghirardi et al., 2022) can help to select patients suitable for minimally invasive surgery. If the disease has already spread intraperitoneally, the already published and validated laparoscopic scoring system (Fagotti et al., 2006) represents a reproducible and cost-effective tool able to assist clinicians in patient’s treatment selection in both upfront and interval debulking surgery. In case of disease recurrence, despite the lack of standardised disease assessment models, laparoscopy in association with imaging techniques appears to be a reliable selection method to identify patients who are the best candidates for surgery to NGR.

Overall, the continuous development of minimally invasive surgical techniques in different treatment settings, in both early and advanced-stage disease, will hopefully provide further interesting research topics on their role at the time of tumour burden assessment.
